# HIV and Bacterial Lipopolysaccharide Activate Inflammasome in Human Oral Keratinocytes: Implications for Aging

**DOI:** 10.1093/geroni/igaf122.2544

**Published:** 2025-12-31

**Authors:** Md Shafayat Jamil, Sofia Lerma, Deepa Roy, Chun Xu, Hansapani Rodrigo, Nirakar Sahoo, Upal Roy

**Affiliations:** The University of Texas Rio Grande Valley, Brownsville, Texas, United States; The University of Texas Rio Grande Valley, Brownsville, Texas, United States; The University of Texas Rio Grande Valley, Brownsville, Texas, United States; The University of Texas Rio Grande Valley, Brownsville, Texas, United States; The University of Texas Rio Grande Valley, Brownsville, Texas, United States; The University of Texas Rio Grande Valley, Edinburg, Texas, United States; The University of Texas Rio Grande Valley, Brownsville, Texas, United States

## Abstract

HIV infection alters the oral microbiome, elevating the presence of Gram-negative bacteria, particularly Porphyromonas gingivalis. P. gingivalis releases lipopolysaccharide (LPS), a powerful immune activator that provokes inflammation in primary human oral keratinocytes, even in antiretroviral therapy-treated people with HIV (PWH). Chronic inflammation is a characteristic of aging, and the activation of inflammasomes is pivotal in the process of inflammation. The Absence of melanoma 2 (AIM2) inflammasome, a DNA-sensing complex, is associated with age-related immunological dysregulation. We propose that HIV sensitizes human oral keratinocytes (HOK) cells to augment AIM2 activation in response to LPS, hence contributing to persistent inflammation and expedited cellular aging. To investigate this hypothesis, HOK was exposed to P. gingivalis LPS at concentrations ranging from 1 ng to 1 μg for 96 hours, and the expression of inflammasome-related genes and proteins was characterized. Concurrently, HOK cells were subjected to HIV exposure for 24 hours and then received LPS treatment for an additional 24 hours. LPS alone increased the expression of AIM2, NLRP3, CASPASE1, CASPASE4, and CASPASE5 in a dose-dependent exposure study. HIV pre-exposure markedly augmented AIM2 activation, resulting in a statistically significant increase in CASPASE 5 and AIM2 gene and protein expression in the presence of LPS. This synergistic effect indicated that HIV prepares HOK for increased inflammasome activation, potentially expediting cellular senescence and immunological aging. Our findings highlight a set of pivotal biomarkers connecting HIV-induced inflammation to cellular senescence, indicating it as a prospective therapeutic target for alleviating inflammation in PWH.

